# Gene Family Expansions Provide Molecular Flexibility Required for Context‐Dependent Species Interactions

**DOI:** 10.1111/ele.70213

**Published:** 2025-09-22

**Authors:** Damian J. Hernandez, Gwendolyn B. Pohlmann, Michelle E. Afkhami

**Affiliations:** ^1^ Department of Biology University of Miami Coral Gables Florida USA; ^2^ Department of Ecology and Evolutionary Biology University of Toronto Toronto Ontario Canada; ^3^ Faculty of Science, Systems Ecology Vrije Universiteit Amsterdam Amsterdam the Netherlands; ^4^ Department of Ecosystem Science & Policy University of Miami Coral Gables Florida USA; ^5^ Department of Art & Art History University of Miami Coral Gables Florida USA

**Keywords:** arbuscular mycorrhizal fungi, context dependency, plant ecology, plant evolution, symbiosis

## Abstract

As environments worldwide change at unprecedented rates during the Anthropocene, understanding context dependency—how species interactions vary depending on environmental context—is crucial. Combining comparative genomics across 42 angiosperms with transcriptomics, genome‐wide association mapping and gene duplication origin analyses, we show for the first time that gene family expansions are important to context‐dependent regulation of species interactions. Gene families expanded in mycorrhizal fungi‐associating plants display up to 200% more context‐dependent gene expression and double the genetic variation associated with mycorrhizal benefits to plant fitness. Moreover, we discover these gene family expansions arise primarily from tandem duplications with > 2‐times more tandem duplications genome‐wide, indicating gene family expansions continuously supply genetic variation, allowing fine‐tuning of context dependency in species interactions throughout plant evolution. Taken together, our results spotlight how widespread gene duplications can provide molecular flexibility required for plant–microbial interactions to match changing environmental conditions.

## Graphical abstract

We explore how the evolution of molecular complexity through gene family expansions is essential for regulating arbuscular mycorrhizal symbiosis in different environmental contexts. Specifically, we use comparative genomics across 42 angiosperms to: (1) demonstrate gene family expansions underpin context‐dependent gene expression regulating symbiosis, (2) genotypic differences in fitness benefits of mycorrhizal symbiosis and (3) identify widespread tandem duplications as a ubiquitous evolutionary mechanism generating these important gene family expansions across angiosperms. Overall, we find that gene family expansions are an essential mechanism for developing complexity needed to regulate context dependency of symbiotic interactions and that discovering how complexity is generated will prove integral to understanding how molecular processes determine when an interaction will be beneficial.

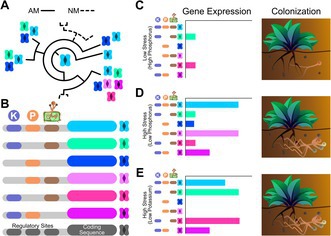

## 1 Introduction

Context dependency is one of the oldest and most pervasive concepts in ecology, affecting organisms across the entire tree of life. For instance, all interactions among species change depending on environmental context (Chamberlain et al. 2014; Liu and Gaines 2022), and this context dependency determines how organisms thrive in their habitats (Chamberlain et al. 2014; Baker et al. 2018), how communities assemble (Bittleston et al. 2020; Gralka et al. 2020) and how ecosystems maintain biodiversity and crucial services (Hagen et al. 2012; Ratzke et al. 2020). While context dependency of species interactions is ubiquitous and well‐documented, the genomic and molecular mechanisms allowing species to integrate environmental cues and modulate their responses to match changing environmental conditions remain largely unknown. Identifying the molecular bases of context dependency will be particularly important in the Anthropocene as humans drive environments to new extremes through climate change, pollution and other escalating stress. Without a clearer molecular mechanistic understanding of context dependency, we are running into new environmental extremes without knowing which species interactions will be resilient to intensifying anthropogenic forces. Here, we discover an essential molecular mechanism underlying context dependency of species interactions in which the generation of genetic complexity through gene family expansions is integral to the genetic plasticity underlying arbuscular mycorrhizal (AM) symbiosis. Specifically, we demonstrate gene family expansions underpin context‐dependent gene expression regulating interactions and genotypic differences in fitness benefits resulting from context‐dependent interactions in an ancient and ecologically significant symbiosis. Further, we identify widespread tandem duplications as a ubiquitous evolutionary mechanism generating these important gene family expansions across angiosperms, linking context dependency of species interactions to their molecular, genomic and evolutionary mechanisms for the first time.

**FIGURE 1:**
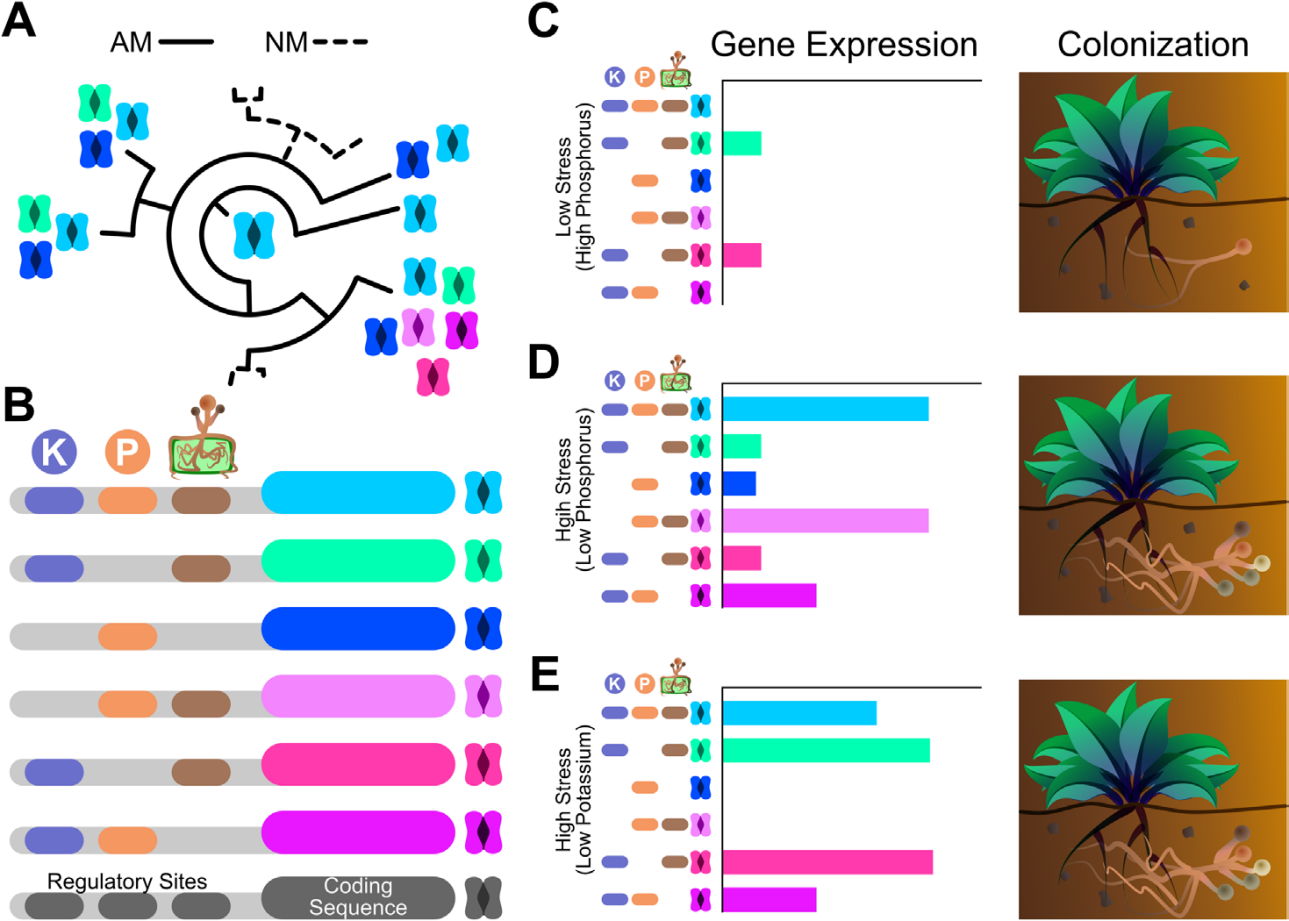
A conceptual framework for how gene families expanded in AM fungi‐associating plants could allow integration of multiple environmental cues. (A) An original ancestor gene (central light blue) that regulates AM symbiosis could evolve into new variants (different colours), resulting in expanded gene families. In contrast, genes can be lost in plants that do not maintain AM symbiosis (dashed line lineages; NM: Non‐mycorrhizal) because there is no selective pressure to keep AM‐relevant genes. (B) The evolution of new genes (colours other than light blue) through expansions can allow for neofunctionalisation/subfunctionalisation through combinatorial coding in which different genes in the family respond to unique combinations of environmental factors like phosphorus availability, potassium availability and mycorrhizal presence. Note that gene family expansions may also integrate environmental cues into regulation of context‐dependent interactions through other pathways not shown here, such as the gain/loss of regulatory target sites on protein sequences. (C–E) Organisms exist in complex and dynamic environments where gene expression changes based on the conditions organisms experience. In diagrams (C–E), we represent the gene family in (B) whose expression promotes AM fungal colonisation but whose genes are differentially expressed in different contexts. The total expression of this gene family determines colonisation, where downregulation of the whole family inhibits colonisation (C) and upregulation promotes it (D, E). Thus, even though the patterns of expression may be different, if the total expression of the gene family remains the same, the outcome for the interaction is the same (e.g., upregulation of different sets of genes within the family in D and E both lead to colonisation). This outcome of similar total expression but different patterns of expression among genes within families may arise from combinatorial coding in the regulatory sites in which some genes respond only to phosphorus and mycorrhizae (pink gene), only potassium and mycorrhizae (cyan and hot pink genes), or a balance between all three (light blue gene).

Organisms navigate complex environments in which conditions frequently change. Success of organisms living under changing conditions often requires the flexibility to efficiently fine‐tune their physiological and molecular responses, which is inherently challenging and likely requires complex regulatory mechanisms. In this study, we demonstrate that the creation/maintenance of large gene families—i.e., related genes descending from a common ancestor—is an essential evolutionary mechanism underpinning how organisms fine‐tune species interactions in response to environmental cues (see Figure 1 for the conceptual model). Large gene families offer opportunities for flexibility in organisms' responses to their environment by creating more points of genetic regulation (i.e., complexity), which allows detailed control of expression of genes with biochemically similar functions under unique combinations of environmental conditions (e.g., *combinatorial coding* in Figure 1; Xie et al. 2018; Müller et al. 2019).

Genetic complexity created through gene family expansions could be essential to the gene expression plasticity species need to respond to different environmental conditions. The mechanisms underlying how gene families expand to build this genetic complexity are critical for how often gene duplications occur and to what extent gene duplications may shape the genetic regulation of species interactions. As gene families expand through processes like whole‐genome and tandem duplications, new genes can then be selected for new functions (e.g., *neofunctionalization* in Figure 1; Young et al. 2011; Mattenberger et al. 2017), which may be important in new environments. For example, in yeast, gene duplication often leads to one copy maintaining its original function and the other developing a new stress‐related function (Mattenberger et al. 2017). While it is well established that gene family expansions are critical for creating genetic complexity underlying novel structures and body plans (Donoghue and Purnell 2005; Young et al. 2011; Furumizu et al. 2015) or adaptation to new environments (Fisher et al. 2018; Hughes et al. 2018), it remains unknown if gene family expansions are important for integrating environmental cues in the context dependency of species interactions. Thus, we determine if the genetic complexity (i.e., greater gene family size) resulting from gene family expansions is important to the context‐dependent gene regulation of AM symbiosis under stressful environments and the genetic variation underlying mycorrhizal benefits to plant fitness.

Additionally, determining the evolutionary origins of gene family expansions is important for understanding whether the regulation of context dependency is dramatically reengineered a few times throughout evolution (e.g., widespread, simultaneous selection across the genome in whole‐genome duplications; Hughes et al. 2018; Qiao et al. 2018, 2019; Bomblies 2020; Almeida‐Silva and Van de Peer 2023) or if the regulation of context dependency is gradually modified over time (e.g., selection on a single gene following tandem duplications; Ponce and Hartl 2006; Fan et al. 2008; Rogers et al. 2015). For example, a whole‐genome duplication in legumes reengineered entire symbiotic pathways and a whole organ devoted to rhizobial mutualism (Young et al. 2011; Young and Bharti 2012). At the other extreme, single‐gene duplications (e.g., tandem duplications) limit effects to individual genes and often impact only one step of a pathway or a few downstream processes (Ponce and Hartl 2006; Fan et al. 2008; Rogers et al. 2015). This form of gene duplication can expand the functional repertoire of a specific class of gene without disrupting broader, essential pathways. These two different evolutionary origins of gene family expansions have other important consequences for macro‐ and micro‐evolutionary trajectories. Whole‐genome duplications, which are rare compared to tandem duplications (Qiao et al. 2019), increase the probability of speciation (Tank et al. 2015; Ren et al. 2018; Walden et al. 2020) and thus also likely reduce the flow of newly duplicated genes into the original species' populations (i.e., new gene variants flow into new species rather than populations of the original species). Alternatively, since tandem duplications cause speciation less frequently, they can provide a continuous source of genetic novelty within species (Hanada et al. 2008; Qiao et al. 2019). Thus, determining which duplication mechanisms are the main drivers of gene family expansions for AM symbiosis genes is critical to predicting, for example, (1) whether entire regulatory pathways undergo changes in selection together and (2) how quickly/whether new genetic variants can spread throughout species populations. In short, discovering the evolutionary origin of duplicated gene families underpinning species interactions is essential for predicting the consequences to species' ecology, evolution and molecular restructuring of context‐dependent processes.

Here, we use one of the most ubiquitous and ecologically important symbioses on Earth—the interaction between AM fungi and plants—to test whether expanded gene families are important to the context dependency of beneficial interactions and how these gene family expansions evolve. The majority of land plants form intracellular associations with AM fungi in which plants provide fungi with carbon in exchange for difficult‐to‐acquire nutrients like phosphorus (Harrison 2005). AM symbiosis is also an ancestral trait of most land plants (Maherali et al. 2016; Strullu‐Derrien et al. 2018), extending back ~450 million years ago when plants were just colonising land (Heckman et al. 2001; Morris et al. 2018). Consequently, AM symbiosis is an excellent model to understand the evolution of mechanisms regulating context dependency because it has a long, shared evolutionary history across nearly the entire plant kingdom but also has replicated, independent losses (e.g., taxa in the family *Brassicaceae*, family *Cyperaceae* and genus *Lupinus*). Together, this makes it possible to identify gene families regulating AM symbiosis with impacts on gene expression and mycorrhizal benefits to plant fitness and determine if gene families regulating AM symbiosis expand through the same evolutionary mechanism across the plant kingdom. We combine comparative genomics across 42 angiosperms with transcriptomics, genome‐wide association study (GWAS) mapping and gene duplication origin analysis to: (1) identify expanded gene families in mycorrhizal‐associating plants, (2) demonstrate that gene family expansions are integral to context‐dependent gene expression and mycorrhizal benefits to plant fitness and (3) discover that gene families expanded in AM‐associating plants primarily grow through tandem duplications. Taken together, our work establishes a molecular eco‐evolutionary principle for context dependency in which the evolution of molecular complexity is essential for regulating symbiotic interactions in different contexts and highlights how gene family expansions and widespread local‐scale duplications are integral to evolving that complexity.

## 2 Materials and Methods

### 2.1 Identifying Gene Families Conserved/Expanded in AM Plants

We identified gene families across 42 angiosperms, 32 AM‐associating and 10 non‐mycorrhizal (NM), that passed our selection criteria. We selected plant species with (1) well‐documented mycorrhizal status, (2) importance as agricultural/research plants and (3) well‐characterised genome annotations. All species in our analyses are widely distributed globally, with distributions spanning continental‐ or global‐scale distributions (Figure S1), and span a wide breadth of angiosperm phylogeny, spanning ANA species and Asterids. We determined mycorrhizal status based on *MycoDB* (Chaudhary et al. 2016) and phylogenetic reconstruction of mycorrhizal status (Maherali et al. 2016). We ensured all genome annotations were constructed through similar processes by limiting genome annotations to RefSeq annotations, which have all been processed through NCBI's Eukaryotic Genome Annotation Pipeline. Using genome annotations that are annotated through the same pipelines is critical for analysing variation in gene family sizes because different algorithms can lead to vastly different numbers of annotated genes due to discrepancies in, for example, cut‐offs for gene detection and criteria for determining what is a gene (Venkatraman et al. 2021; Weisman et al. 2022). We excluded plants that can symbiose with both ectomycorrhizal and AM fungi to avoid identifying genes involved in the phylogenetically distinct and unrelated ectomycorrhizal symbiosis. By using these criteria, we balance the need to optimise phylogenetic representation, limit the effects of using different genome annotation methods and use high‐quality genomes to conduct a rigorous comparative genomics analysis. We then grouped genes into ‘orthogroups’, all extant genes descending from a single gene, with *OrthoFinder* using default parameters, and only selected the longest isoform for each gene, as recommended by the authors (v2.2.6; Emms and Kelly 2015, 2019).

To identify gene families conserved in AM plants, we compared gene family sizes between AM and NM plants using Mann–Whitney *U*‐tests for each gene family followed by multiple hypothesis correction (Benjamini‐Hochberg). We repeated this selection with phylogenetic corrections, and all downstream analyses resulted in qualitatively similar outcomes (Supporting File S1) due to high overlap between both selection strategies (~90% of gene families are shared between both strategies). Note that AM‐expanded gene families could result from both selection for the maintenance and the enlargement of gene families over evolutionary time scales. While it is not computationally possible to differentiate between maintenance and expansion in this comparative genomics analysis, results from our gene duplication origin analyses strongly support AM‐expanded gene families arising from the enlargement of gene families rather than maintenance. To verify that these putative AM‐expanded gene families are involved in AM symbiosis, we validated our selection by (1) loss of known AM‐symbiosis genes in NM plants (Figure S2a) and (2) determining if AM‐expanded gene families are more likely to be differentially expressed in plants grown with AM fungi relative to plants that were not (Figure S2b).

### 2.2 Determining if Larger AM‐Expanded Gene Families Have More Context‐Dependent Expression

To determine if larger AM‐expanded gene families are hotspots of context‐dependent expression underlying gene expression responding to AM symbiosis and environmental stress, we analysed RNA‐sequencing datasets of plants grown with/without mycorrhizal fungi and with/without an environmental stressor. We queried the NCBI Gene Expression Omnibus and BioProject databases for the search terms ‘arbuscular’, ‘rhizophagus’, ‘funneliformis’, ‘glomus’ and ‘mycorrhizal’. We manually reviewed all search hits for datasets with plants grown in a factorial design of +/− mycorrhizal fungi and +/− environmental stimuli. We also queried Google Scholar for the terms ‘AM symbiosis’, ‘RNA‐sequencing mycorrhizae’, ‘AM symbiosis stress’, ‘Rhizophagus stress’, ‘AM symbiosis drought’, etc.

For each factorial dataset, we assigned genes to the gene families constructed above and quantified their expression (see Supporting Text S1 for details). If data were provided as raw sequencing data instead of gene count tables, we processed raw FASTQ files and quantified expression using the *STAR* aligner (Dobin et al. 2013). Scripts used to download and process raw sequencing reads are available in the Zenodo repository associated with this manuscript (DOI: 10.5281/zenodo.16945424). For each dataset, we evaluated which genes showed context‐dependent expression in response to mycorrhizal fungi and the environmental stimuli using *DESeq2* (Love et al. 2014) by comparing fully factorial models including an interaction term with a model missing the interaction term (likelihood ratio tests; FDR < 0.05). Significant interaction terms indicate non‐additive, context‐dependent gene expression.

To determine if larger AM‐expanded gene families are disproportionately expressed context‐dependently, we compared the observed strength of the relationship between gene family size and the proportion of context‐dependently expressed genes to relationships in 10,000 permuted subsamples of the whole genome (see Supporting Text S1 for details). We calculated *p*‐values as the number of random subsamples that had a stronger (i.e., higher *⍴*) or equal relationship than the observed divided by 10,000.

### 2.3 Determining if Larger AM‐Expanded Gene Families Have More Fitness‐Associated Natural Genetic Variation

To determine if larger AM‐expanded gene families are disproportionately associated with mycorrhizal effects on plant fitness, we performed a GWAS on 212 naturally occurring *M. truncatula* populations (Medicago HapMap Project n.d.) and quantified how much genetic variation is associated with changes in pod counts (i.e., an important fitness metric for this annual plant) between plants grown with and without AM fungi (see Afkhami et al. 2021 for experiment details). We identified SNPs across the 212 genotypes, then filtered to remove SNP sites with minor allele frequencies less than 5% and SNPs for which more than 10% of populations had no available sequencing data for that position (*bcftools*; Li 2011). We imputed missing data with the most common allele for that site (*impute* function, *LEA* package).

We used latent factor mixed models (LFMMs) to identify SNPs significantly associated with benefits to plant fitness from associating with AM fungi (*lfmm2* function, *LEA* package; Caye et al. 2019). We identified 6–8 latent factors for the LFMM models based on groupings in the genetic data: an elbow plot of a principal component analysis (Figure S3), an elbow plot of cross‐entropy criteria from admixture coefficient analyses (Figure S4) and a visual assessment of *Q*‐matrices from the best ‘run’ in the admixture coefficient analyses for different numbers of ancestral populations (Figure S5). We represent results in the manuscript using seven latent factors because that is the middle of the range, but results are consistent with six or eight latent factors. We corrected the *p*‐values of each LFMM for each SNP using a Benjamini‐Hochberg correction. We then asked if larger AM‐expanded gene families had more genetic variation associated with mycorrhizal effects on plant fitness (i.e., pod counts) than smaller gene families. We concatenated the gene sequences of all genes within each gene family and determined what percentage of that concatenated gene family had genetic variation associated with changes in pod counts between plants inoculated with AM fungi and those that were not. We performed a Spearman's correlation analysis to determine the strength of the relationship between gene family size and the percentage of a gene family's genomic sequence with fitness‐associated variation and compared this observed relationship to 10,000 random subsamples from the whole genome.

### 2.4 Identifying the Duplication Mechanisms Driving Growth of AM‐Expanded Gene Families

To identify which duplication mechanisms are driving growth in AM‐expanded gene families, we reconstructed the *duplicate_gene_classifier* function from the *MCScanX* package (Wang et al. 2012) in *R* to leverage the use of gene families for quantifying different kinds of gene duplication mechanisms (tandem, proximal, dispersed and whole‐genome/segmental duplications; see Supporting Text S1 for details). We used the *MCScanX_h* function in the *MCScanX* package to identify whole‐genome/segmental duplications. If genes were indicated as products of different duplication mechanisms, we chose the mechanism based on *MCScanX*'s *duplicate_gene_classifier* logic: WGD > tandem > proximal > dispersed > singleton. To determine if AM‐expanded gene families disproportionately arise from any one type of duplication event, we calculated the observed proportion of genes in AM‐expanded gene families that belonged to each type of duplication for each species. We compared those observed proportions to 10,000 random subsamples of all gene families in each species (the number of randomly subsampled gene families is equal to the number of AM‐expanded gene families in that species) and corrected for multiple comparisons (Benjamini‐Hochberg).

### 2.5 Data Availability

All analyses were performed in *R* (v 4.1.2; R Core Team 2025) with the packages listed in Supporting File S1 unless otherwise indicated. All original data are publicly available and can be found as indicated in Supporting File S1 or in our Zenodo repository (DOI: 10.5281/zenodo.16945424). Computational scripts, processed data and analyses are fully available in Zenodo. Processed data are also available in Supporting File S1. An extended Methods section with further detail is available in Supporting Text S1.

## 3 Results

**FIGURE 2:**
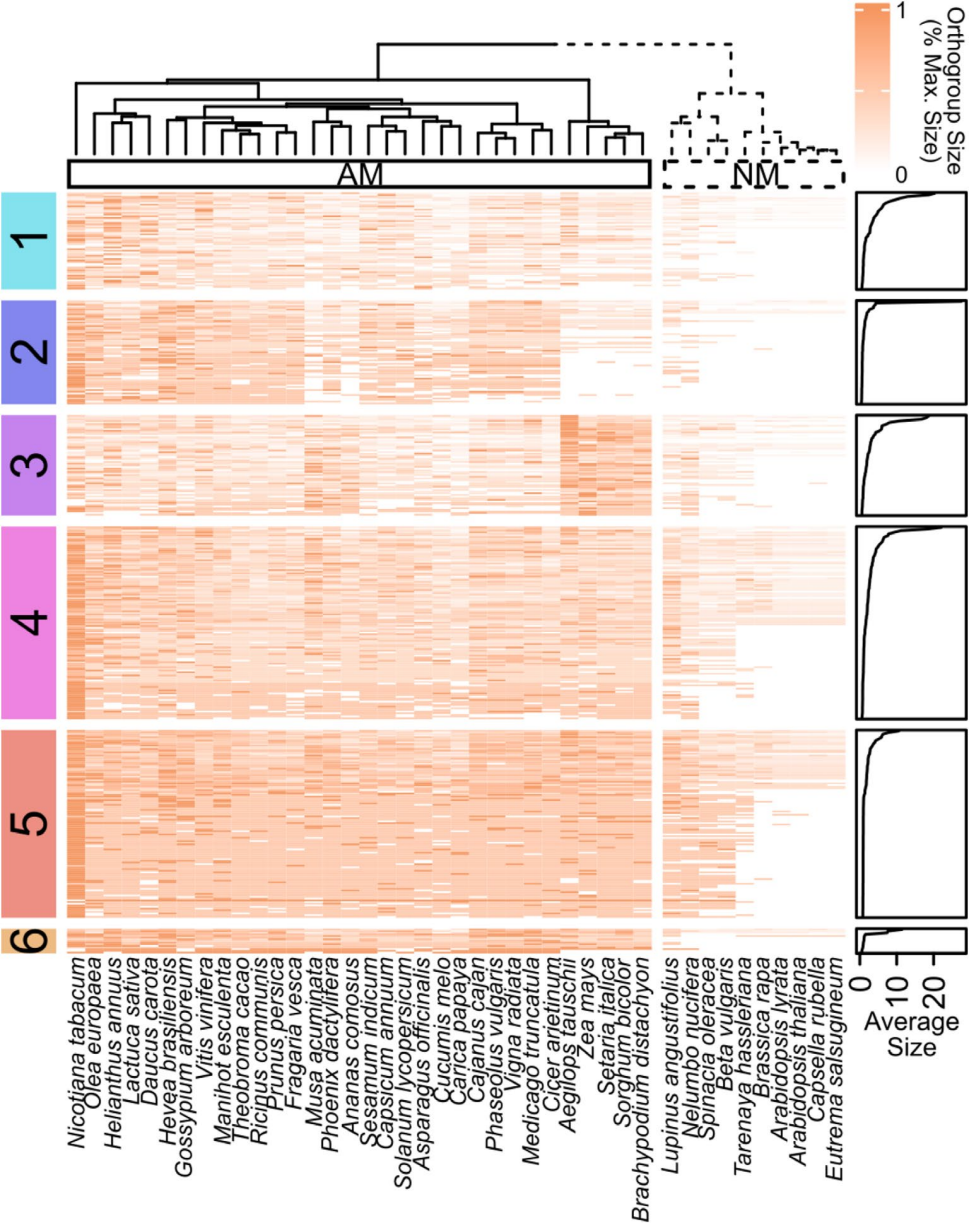
Comparative genomics identified 429 AM‐expanded gene families as candidates for context‐dependent regulation of the widespread symbiosis between mycorrhizal fungi and plants. Our comparative genomic analyses identified 429 gene families that were significantly larger in AM plants across 32 arbuscular mycorrhizal (AM, left) and 10 non‐mycorrhizal (NM, right) plant species (Mann–Whitney *U*‐Tests, Benjamini‐Hochberg correction). We find interesting co‐expansion patterns of gene families across AM plants and lineage‐specific patterns in grasses (see Supporting Text S2 for details). For example, Cluster 1 is highly enriched in genes localised to membranes and arbuscules, such as the EIX2 fungal receptors and Vapyrin‐like proteins that may organise arbuscule development. Each row represents a gene family, with darker colours indicating larger gene families. Plant species (columns) are clustered within the heatmap using unsupervised hierarchical clustering (Ward's D2 linkage, Euclidean distances) on gene family sizes, which neatly split AM and NM plants into two distinct groups. We used k‐means clustering on gene families (rows) to identify six groups (coloured labels on left). To allow for comparison of gene families across rows, values within each cell were normalised across the row (values between 0 and 1).

To identify gene families expanded in AM plants, we used comparative genomic analyses across 42 plant species (32 AM, 10 NM) to determine which gene families were significantly larger in plants that can symbiose with AM fungi (Supporting Text S1; Maherali et al. 2016; Strullu‐Derrien et al. 2018). We identified 429 gene families (1.5% of 28,756 families) expanded in AM plants (Figure 2) and found several lines of evidence supporting the role these AM‐expanded gene families play in AM symbiosis. First, 70% of genes in the Common Symbiosis Pathway (a pathway required for establishing AM symbiosis; Oldroyd 2013) are in AM‐expanded gene families, which is 47 times greater than the null expectation of 1.5% of all gene families (*p* < 0.0001, binomial test; Figure S2a). In fact, only the three nucleoporins *SEH1*, *NUP85* and *NUP133* were not in AM‐expanded gene families, suggesting that the nuclear transport organised by these proteins may be essential for AM establishment but not unique to it, unlike processes organised by genes mediating nuclear calcium spiking (*CASTOR* and *DMI1/POLLUX*), inducing hyphopodia formation (*RAM2*), and reprogramming transcription (*DMI3/CCamK*, *IPD3/CYCLOPS*, *RAM1* and *NSP1*), which may be essential and unique to AM symbiosis or recently co‐opted for other symbioses. Second, we find genes within AM‐expanded gene families are 63% more likely to be differentially expressed than the rest of the genome when plants are grown with AM fungi compared to when they are not (*p* < 0.0001, binomial test; Figure S2b; Afkhami and Stinchcombe 2016), thus supporting expression of genes in AM‐expanded gene families as an important regulatory target for plants during AM symbiosis. Our analyses also revealed interesting co‐expanding groups of gene families, providing insight into co‐regulated processes in AM symbiosis across angiosperms and modifications to symbiosis in grasses (see Supporting Text S2, Supporting File S1). Taken together, our comparative genomics analysis identifies gene families likely functioning in AM symbiosis across plants whose last common ancestor was ~160–200 million years ago (Li et al. 2019).

We then asked whether larger AM‐expanded gene families are hotspots of context‐dependent regulation and mycorrhizal benefits to plant fitness. If gene family expansions underpin context‐dependent regulation of AM symbiosis, plants should disproportionately regulate genes in larger AM‐expanded gene families in response to the combination of AM symbiosis and environmental stress rather than these two factors separately (i.e., significant interactive effects of mycorrhizal fungi and environment indicate context‐dependent gene expression). Also, if larger AM‐expanded gene families are important for context‐dependent regulation of AM symbiosis and, consequently, fitness outcomes in associating plants, then larger gene families should contain more genetic variation associated with mycorrhizal‐derived fitness benefits than the rest of the genome. Therefore, we (1) conduct a context‐dependent expression analysis to determine if plants disproportionately regulate the expression of genes in larger gene families during AM symbiosis in stressful conditions and (2) perform a GWAS to determine if larger gene families are hotspots of genotypic differences in mycorrhizal‐derived benefits to plant fitness.

**FIGURE 3:**
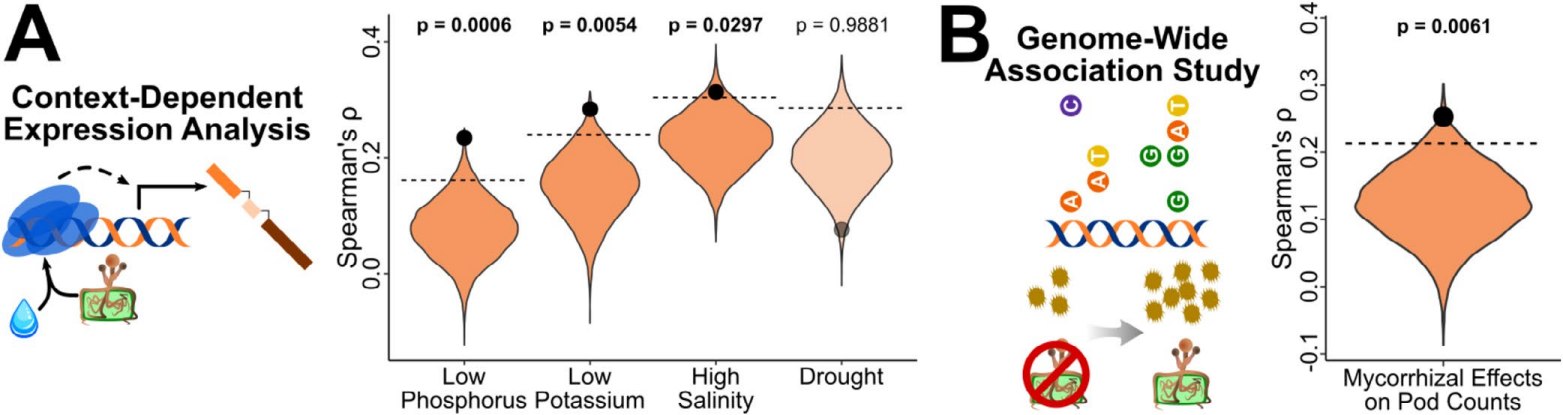
Larger AM‐expanded gene families are hotspots of context‐dependent gene regulation and mycorrhizal benefits to host plant fitness. (A) Our transcriptomic analyses discovered that larger AM‐expanded gene families have 40%–200% more context‐dependent expression in response to soil chemical stress and mycorrhizal presence than expected by chance. We quantified the strength of the relationship between each gene family's size and the proportion of genes in each family that were context‐dependently expressed (Spearman's Correlation Coefficients; black dots) and compared these observed relationships to the null expectation generated by permutational analysis with 10,000 random subsamples of all gene families (violins). (B) Our genome‐wide association study (GWAS) also identified larger AM‐expanded gene families as genetic hotspots for mycorrhizal benefits to plant fitness, harbouring ~100% more associated SNPs than the rest of the genome. To determine if larger AM‐expanded gene families contain more genetic variation underlying naturally‐occurring variation in plant fitness benefits from AM fungi, we determined the number of SNPs associated with increases (or decreases) in pod counts in 212 *M. truncatula* genotypes grown with versus without AM fungi using the GWAS. We then quantified the relationship between the normalised SNP density of each gene family (i.e., number of significantly associated SNPs divided by the entire sequence length of each gene family which accounts for variation in the number of genes and gene lengths in different families) and related normalised SNP density to gene family size using a Spearman's correlation test (observed coefficient displayed as black dot). To determine if those observed relationships were significant, we compared the observed coefficient to that from 10,000 random subsamples of all gene families (violin). In (A) and (B), the dashed lines represent the 95% significance cut‐offs; actual coefficients (black dots) above these lines indicate a significantly stronger relationship between gene family size and context‐dependent expression or between gene family size and genetic variation than expected by chance. Faded points and violins represent non‐significant relationships.

First, we found that larger AM‐expanded gene families had up to 200% greater context‐dependent expression under soil chemical stress than the rest of the plant genome (Figure 3a), indicating that gene expression plasticity facilitated by expanded gene families provides an important genomic basis for context‐dependent regulation. We analysed RNA‐sequencing datasets of plants grown in factorial experiments with/without AM fungi and with/without a stressor—either low phosphorus, low potassium, high salinity, or drought (Calabrese et al. 2019; Garcia et al. 2017; Ren et al. 2019; Recchia et al. 2018). We then determined if larger AM‐expanded gene families had significantly higher proportions of genes whose expression is context‐dependent. To do this, we quantified the relationship between gene family size and proportion of genes in AM‐expanded gene families with significant context‐dependent expression compared to the same relationship in 10,000 random subsamples of all gene families. We use the proportion (instead of the total number) of differentially expressed genes to avoid a size bias (see Supporting Text S1). We found plants disproportionately target larger AM‐expanded gene families for context‐dependent regulation under soil chemical stress, with 200%, 86% and 41% greater context‐dependent expression of these gene families than expected by chance in response to low phosphorus, low potassium and high salinity stress, respectively (*p*‐values from permutations: *p* = 0.0006, *p* = 0.0054 and *p* = 0.0297; Figure 3a), indicating the gene expression plasticity facilitated by gene family expansions is important for context‐dependent regulation under soil chemical stress. Interestingly, under drought, larger gene families do not contain more context‐dependent expression (observed Spearman's *ρ*: 0.076; mean random *ρ*: 0.199; *p* = 0.9881; Figure 3a) but may actually contain less (*p* = 0.0119; 61% less than random on average), matching the importance of gene family contraction for drought stress responses in *Arabidopsis thaliana* and the grass *Pseudoroegneria libanotica* (Monroe et al. 2018; Zhai et al. 2024; discussed further in Supporting Text S2). This dichotomy between soil chemical stressors and drought potentially points to two distinct molecular syndromes driving the ecological strategies plants use to regulate AM symbiosis during persistent stress lasting years/decades versus seasonal/episodic stress (see Supporting Text S2). In both cases, our transcriptomic analyses demonstrate that gene family size is an essential component of how plants regulate gene expression when integrating environmental context into AM symbiosis.

Second, larger AM‐expanded gene families harbour two times more genetic variation associated with mycorrhizal benefits to plant fitness than the rest of the genome in the model legume *Medicago truncatula* , highlighting how gene family expansions can underpin significant intraspecific variation in mycorrhizal benefits. Motivated by our finding that plants disproportionately target larger gene families for regulating gene expression during AM symbiosis under soil chemical stress, we evaluated whether these AM‐expanded gene families disproportionately impact mycorrhizal benefits to plant fitness by conducting a GWAS across 212 *M. truncatula* genotypes (Stanton‐Geddes et al. 2013; Medicago HapMap Project n.d.). The GWAS identified genetic variation—SNPs, specifically—linked to mycorrhizal effects on the key plant fitness metric of seed pod counts by assessing changes to seed pod counts in plants grown with AM fungi compared to uninoculated plants (Afkhami et al. 2021). We used permutational tests to determine if larger AM‐expanded gene families contained more fitness‐associated SNPs than expected (compared to 10,000 random subsamples of the entire genome). Larger AM‐expanded gene families contained approximately double the genetic variation underpinning mycorrhizal effects on fitness than expected by chance (observed Spearman's *⍴* = 0.252, mean random *⍴* = 0.125 ± 0.001; *p* = 0.0061; Figure 3b), indicating these gene families are hotspots for genetic variation underlying mycorrhizal effects on plant fitness. This emphasises that in addition to being focal points for soil chemistry‐driven regulation, larger AM‐expanded gene families are an important source of the genetic variation determining mycorrhizal benefits.

Taken together, these results raise the question: how are these gene families expanding? Whether expansions occur through massive duplication events like whole‐genome duplications or through smaller, single‐gene duplications can have profound consequences for how frequently new genes are created (Qiao et al. 2019) and the extent to which molecular pathways are reengineered (Freeling 2009; Almeida‐Silva and Van de Peer 2023). Consequently, we sought to determine which duplications were the main drivers of growth for AM‐expanded gene families.

**FIGURE 4:**
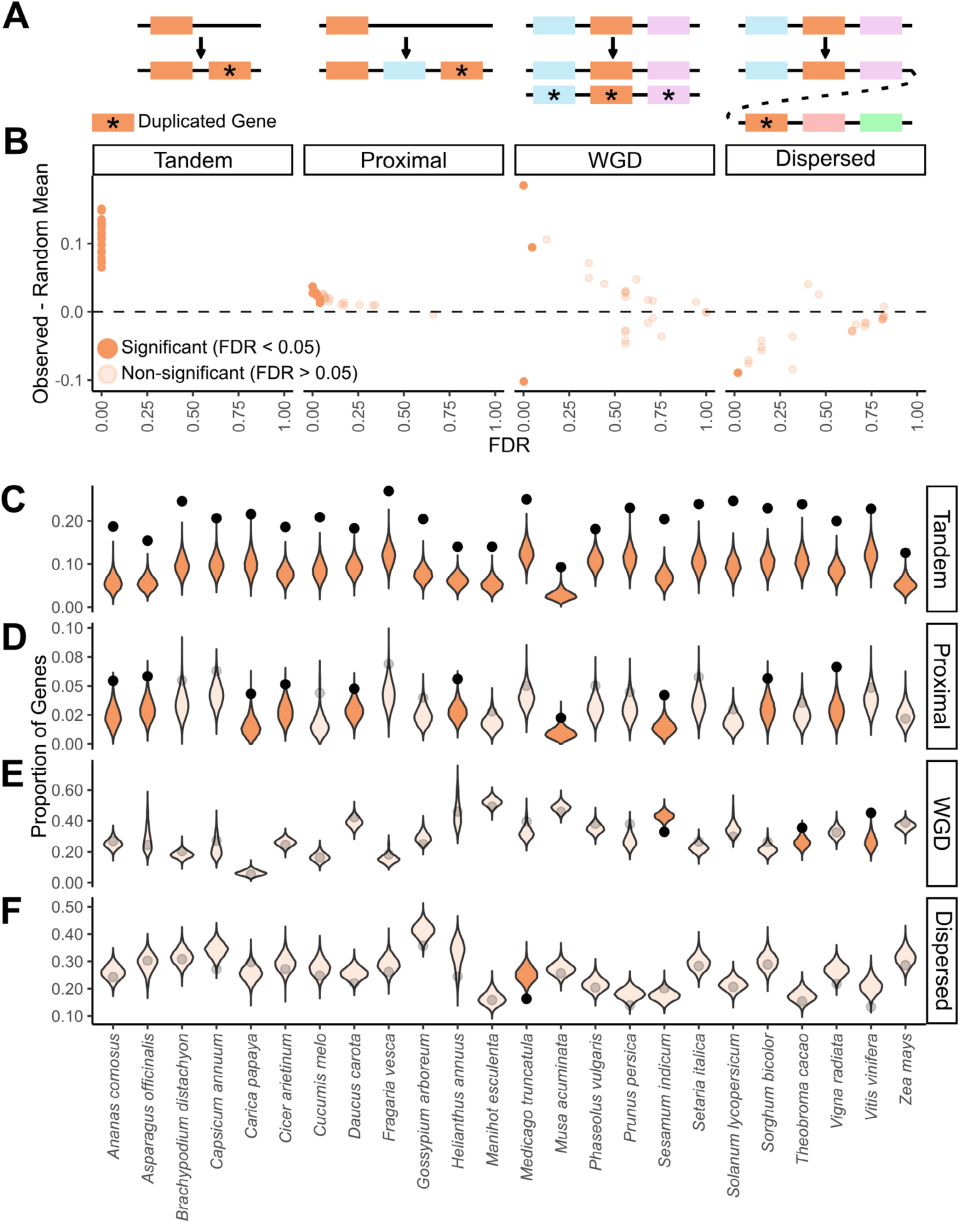
Tandem duplications are the main driver of growth in AM‐expanded gene families across all angiosperm species examined. (A) Cartoon schematic of four duplication classes evaluated—tandem, proximal, segmental/whole‐genome duplication (WGD) and dispersed duplications—with focal genes in orange and duplicate genes marked by asterisks. (B) Frequency of different duplication events in AM‐expanded gene families compared to the whole genome across 24 angiosperms. Each point represents one angiosperm species. The *Y*‐axis displays the difference between the actual frequency of that type of duplication in AM‐expanded gene families and the null expectation (averages from 10,000 randomisations; displayed as violin distributions in C–F). The *X*‐axis shows the Benjamini‐Hochberg‐corrected *p*‐values (FDR) of each comparison between the actual frequency in AM‐expanded gene families and the frequencies in the random subsamples. Significant points above the dashed line (the dashed line represents no difference between observed and random mean) represent significant enrichment of that duplication type in that species, and significant points below the dashed line represent depletion. (C–F) Violin plots showing which types of duplication occur significantly more (or less) often in AM‐expanded gene families than expected by chance for each of the 24 plant species. Black points represent the proportion of genes in AM‐expanded gene families originating from tandem (C), proximal (D), whole‐genome/segmental (E) and dispersed (F) duplications found in our analyses. Violin distributions display the null expectations (i.e., distribution of genes that were classified as each duplication type in 10,000 random subsamples of all gene families) for all 24 tested species. Unfaded points and violins are significant relationships (FDR < 0.05; observed proportion of duplications outside of 95% of the null distribution), and faded points and violins are non‐significant.

We find that AM‐expanded gene families grow > 2 times as often through tandem duplications (duplication of genes immediately adjacent to each other on a chromosome) as the rest of the genome across all plant species tested (Figure 4), indicating that tandem duplication is the primary evolutionary driver of expanding gene families underpinning context dependency of this symbiosis. We conducted a gene duplication origin analysis using a customised *MCScanX* pipeline (DOI: 10.5281/zenodo.16945424; Wang et al. 2012) and quantified the frequency of different types of duplications in AM‐expanded gene families across 24 plant species with high‐quality chromosome scaffolds (results are also consistent across all 32 AM‐associating species; Figure S6). We then compared those observed frequencies with 10,000 random subsamples of gene families from each species' genome (Methods). In all AM‐associating plant species (24/24, FDR < 0.05), genes in AM‐expanded families disproportionately originate from tandem duplications (2.28 ± 0.08 times more common than random; Figure 4b,c). This is further supported by modest enrichment of proximal duplications (duplications with 1–20 genes between duplicates; Supporting Text S2) in 42% of plant species (10/24 significant, and all species except *Zea mays* show the same trend; Figure 4b,d) because many proximal duplications are older tandem duplications that have been interrupted by events like gene insertion (Wang et al. 2012; Qiao et al. 2019). Dispersed and whole‐genome/segmental duplications are negligible drivers of growth in AM‐expanded gene families (0% and 8.33% of species, respectively; Figure 4e,f). The enrichment for tandem duplications among AM‐expanded gene families is particularly interesting because genes arising from tandem duplications are far less common than whole‐genome or dispersed duplications in plants (Figure 4; ~3–10 times less common than WGD or dispersed duplications in most plants; Wang et al. 2012; Qiao et al. 2019; The Arabidopsis Genome Initiative 2000). Thus, the enrichment for tandem duplications and the relative rarity of tandem duplication as a mechanism in plants suggest that AM‐expanded gene families are experiencing unique selection pressures compared to most duplicated genes in the plant genome. Taken together, these results demonstrate that tandem duplication is the primary evolutionary source of AM‐expanded gene families, highlighting that local, small‐scale duplications have profound impacts on context‐dependent regulation of AM symbiosis across angiosperms.

## 4 Discussion

Our study demonstrates for the first time that (1) gene family expansions are an important mechanism through which species build molecular complexity needed to regulate symbiotic interactions in changing environmental conditions, and (2) tandem duplications are the main evolutionary mechanism facilitating gene expression plasticity and gene family expansions underlying context‐dependency. Using the widespread and ecologically important symbiosis between plants and AM fungi as a model, we found that expanded gene families are hotspots for both context‐dependent genetic regulation and genetic variation underpinning organismal fitness. Surprisingly, expanded gene families experienced up to 200% more context‐dependent changes in molecular regulation of the symbiosis in response to soil chemistry changes and twice as many fitness‐associated SNPs as the rest of the genome, emphasising the importance of this mechanism for providing molecular plasticity required for context‐dependency and modulating fitness benefits of symbiosis. Our results also highlight how influential micro‐evolutionary processes—widespread tandem duplication—can shape how organisms regulate their symbioses. In fact, expanded gene families regulating AM symbiosis primarily grew through tandem duplications across all 32 tested plant species, with > 2 times more tandem duplications in AM‐expanded gene families than the rest of the genome. Below, we discuss (1) how evolutionary processes driving gene family expansions shape species' ecologies in changing environments and (2) how integrating molecular biology into ecological frameworks generates valuable mechanistic understanding of complex ecological processes.

Our research highlights tandem duplications as a key source of genetic variation for context‐dependent regulation of symbioses. Tandem duplications continuously occur throughout plant evolution (Qiao et al. 2019) without the high likelihood of speciation resulting from more disruptive processes like whole‐genome duplications (Tank et al. 2015; Baduel et al. 2018; Ren et al. 2018; Walden et al. 2020). Thus, tandem duplications can provide new genes that spread through species' populations and increase the functional repertoire for regulating context dependency *within* species (i.e., microevolution). This contrasts with whole‐genome duplications which are also important for creating evolutionary novelty but can generate macroevolutionary changes like speciation and radiation (e.g., as in legumes, angiosperms, etc.; Tank et al. 2015; Baduel et al. 2018; Almeida‐Silva and Van de Peer 2023). These macroevolutionary changes can in turn reduce the flow of newly duplicated genes back into the original species' populations. Consequently, tandem duplications may be important for generating variation in gene copy numbers within a population. Our results support this evolutionary mechanism by discovering enrichment of context‐dependent gene regulation and fitness‐associated variation in larger gene families. In short, our work highlights the importance of tandem duplications as a main driving force in the context‐dependent regulation of AM symbiosis. We hypothesise tandem duplications and gene family expansions will be a common mechanism for context‐dependent regulation of species interactions (particularly other symbioses) across the tree of life and advocate for future work across a wide range of species interactions.

Our work also illustrates how integrating molecular biology with ecological theory can meaningfully expand our understanding of context‐dependent mechanisms and their biological consequences. Historically, researchers of context dependency have built value‐based models (e.g., Biological Market Theory, Stress Gradient Hypothesis, etc.; Noë and Hammerstein 1995; Bertness and Callaway 1994) in which each species provides a resource whose value shifts based on the environmental context (e.g., mycorrhizal‐derived phosphorus has more value in low‐phosphorus soils than in high‐phosphorus soils). These value‐based models provide easy‐to‐understand, ecologically meaningful reasons why interactions change. Genome‐wide approaches, like we use here, provide an important complementary perspective by generating holistic insight into how species determine the value of resources in different environments. For example, by using comparative genomics, we identified unique gene family co‐expansion in specific plant lineages that highlight likely changes in how species value certain resources. For instance, several co‐expanding gene families involved in starch/carbon metabolism are highly reduced in grasses when compared to other plants (e.g., OG0013009 involved in glycogen metabolism and OG0013006 involved in sucrose catabolism; Supporting File S1), suggesting the value of carbon in AM symbiosis is altered in grasses and emphasising the importance of generating grass‐specific models of carbon‐phosphorus trade in AM symbiosis rather than inferring trade dynamics from symbioses with other plant lineages (Supporting Text S2). To identify more lineage‐specific differences in the genetic basis of ecological mechanisms like resource valuation, we advocate for expanding the phylogenetic representation of species in large databases undergoing shared genome annotation pipelines. For example, Brassicaceae species are over‐represented NM plants in many genomic databases. More annotations for other large NM lineages like Caryophyllales, Lamiales and Cyperaceae would be ideal for identifying novel genetic processes underlying AM symbiosis by including species with distinct evolutionary histories, environmental preferences and times since their ancestors lost the ability to form AM symbioses.

Further, our study highlights a pertinent frontier at the intersection of mechanistic understanding of context dependency and global change biology in which gene family expansions can create genetic variation within species that is used to regulate their interactions and potentially better navigate new, more stressful extremes in the Anthropocene. However, our study showed the importance of expanded gene families as a mechanism for modulating beneficial symbioses may depend on the type of stressor, with different mechanisms for persistent and seasonal/episodic stresses, such that the complexity generated by gene family expansions may be more beneficial under persistent stressors like low nutrient availability but disadvantageous under episodic stressors like drought (full discussion of the relationship between genetic complexity and temporal structure of stressors in Supporting Text S2). Future work exploring how molecular complexity relates to the temporal characteristics of stressors would be useful for linking genomic data to the susceptibility of different species to their new environmental conditions in the Anthropocene (e.g., more intense/frequent droughts, warmer temperatures, etc.). These genomic experiments linking genetic variation and molecular complexity to fitness consequences of mycorrhizal fungi in changing environments are possible in the near future by leveraging existing genetic databases of other model AM‐associating plant species like *Zea mays* and *Triticum aestivum* in population genetic analyses like genome‐wide association mapping and genotype‐environment associations. Additionally, the importance of large gene families increases the complexity of genetic networks and, consequently, the relative importance of epistasis (Jiang et al. 2011). As a result, quantitative genetic analyses like line cross analyses between inbred lines with different gene copy numbers for gene families of interest may be valuable to understand how gene duplications reorganise gene/protein interaction networks in context dependency. Overall, we predict a flourishing of innovative molecular perspectives applied to context dependency will be possible as massive collaborative efforts to expand molecular databases come online (e.g., the 10,000 Plant Genome Project for plants, i5k for insects, the Refuge 2022 Consortium for corals, the 1000 Fungal Genome Initiative, etc.; Cheng et al. 2018; Grigoriev et al. 2014; Levine 2011; Voolstra et al. 2015), thus highlighting how the current state of the field is at the cusp of linking molecular mechanisms to large‐scale ecological consequences.

In summary, gene family expansions are an essential mechanism for developing the complexity needed to regulate the context dependency of symbiotic interactions. We demonstrate this in the widespread AM symbiosis. Specifically, we show how plants disproportionately regulate the largest AM‐expanded gene families and that these gene families contain almost double the amount of genetic variation associated with mycorrhizal‐derived plant fitness. We also find a ubiquitous evolutionary mechanism through which gene family expansions occur in this interaction, highlighting how small, local duplications may be essential to the microevolution of context‐dependent regulation of interactions. Our work demonstrates that complexity is not obfuscating the molecular mechanisms underlying context dependency but is actually an important tool underlying context‐dependent regulation of interactions. Consequently, we should embrace the complexity of context dependency and explore where and when complexity originates as species interactions evolve. Discovering how complexity is generated will prove integral to understanding not only under which conditions an interaction will be beneficial or harmful but also how processes occurring at a molecular level cascade to shape interactions between entire species.

## Author Contributions

Conceptualisation and Supervision: D.J.H., M.E.A. Methodology and Funding acquisition: D.J.H., G.B.P. Visualisation: D.J.H. Writing: D.J.H., G.B.P., M.E.A.

## Conflicts of Interest

The authors declare no conflicts of interest.

## Peer Review

The peer review history for this article is available at https://www.webofscience.com/api/gateway/wos/peer‐review/10.1111/ele.70213.

## Supporting information


**Data S1:** ele70213‐sup‐0001‐Supinfo.docx.

**Data S2:** ele70213‐sup‐0002‐SupplementaryData1.xlsx.

## Data Availability

Raw data are publicly available and are linked to the public databases from which they were retrieved in a Zenodo repository (DOI: 10.5281/zenodo.16945424). Scripts to analyse the data and processed data for/from statistical analyses are available in Zenodo (e.g., scripts and outputs for analysing context‐dependent expression, genetic variation and gene duplication). Metadata and processed data used in statistical analyses are also available in Supporting File S1. The data in Supporting File S1 is also available in the Zenodo archive, but we include this file so others do not need to download the entirety of our project when they may only want/need a specific table.

## References

[ele70213-bib-0001] Afkhami, M. E. , M. L. Friesen , and J. R. Stinchcombe . 2021. “Multiple Mutualism Effects Generate Synergistic Selection and Strengthen Fitness Alignment in the Interaction Between Legumes, Rhizobia and Mycorrhizal Fungi.” Ecology Letters 24: 1824–1834. 10.1111/ele.13814. 34110064

[ele70213-bib-0002] Afkhami, M. E. , and J. R. Stinchcombe . 2016. “Multiple Mutualist Effects on Genomewide Expression in the Tripartite Association Between *Medicago truncatula* , Nitrogen‐Fixing Bacteria and Mycorrhizal Fungi.” Molecular Ecology 25, no. 19: 4946–4962. 10.1111/mec.13809. 27543961

[ele70213-bib-0003] Almeida‐Silva, F. , and Y. Van de Peer . 2023. “Whole‐Genome Duplications and the Long‐Term Evolution of Gene Regulatory Networks in Angiosperms.” Molecular Biology and Evolution 40, no. 7: msad141. 10.1093/molbev/msad141. 37405949 PMC10321489

[ele70213-bib-0004] Baduel, P. , S. Bray , M. Vallejo‐Marin , F. Kolář , and L. Yant . 2018. “The “Polyploid Hop”: Shifting Challenges and Opportunities Over the Evolutionary Lifespan of Genome Duplications.” *Frontiers in Ecology and Evolution* 6. Accessed March 15, 2023. https://www.frontiersin.org/articles/10.3389/fevo.2018.00117.

[ele70213-bib-0005] Baker, D. M. , C. J. Freeman , J. C. Y. Wong , M. L. Fogel , and N. Knowlton . 2018. “Climate Change Promotes Parasitism in a Coral Symbiosis.” ISME Journal 12, no. 3: 921–930. 10.1038/s41396-018-0046-8. 29379177 PMC5864192

[ele70213-bib-0006] Bertness, M. D. , and R. Callaway . 1994. “Positive Interactions in Communities.” Trends in Ecology & Evolution 9, no. 5: 191–193. 10.1016/0169-5347(94)90088-4. 21236818

[ele70213-bib-0007] Bittleston, L. S. , M. Gralka , G. E. Leventhal , I. Mizrahi , and O. X. Cordero . 2020. “Context‐Dependent Dynamics Lead to the Assembly of Functionally Distinct Microbial Communities.” Nature Communications 11, no. 1: 1440. 10.1038/s41467-020-15169-0. PMC7080782 32188849

[ele70213-bib-0009] Bomblies, K. 2020. “When Everything Changes at Once: Finding a New Normal After Genome Duplication.” Proceedings of the Royal Society B: Biological Sciences 287, no. 1939: 20202154. 10.1098/rspb.2020.2154. PMC7739491 33203329

[ele70213-bib-0011] Calabrese, S. , L. Cusant , A. Sarazin , et al. 2019. “Imbalanced Regulation of Fungal Nutrient Transports According to Phosphate Availability in a Symbiocosm Formed by *Poplar*, *Sorghum*, and *Rhizophagus irregularis* .” Frontiers in Plant Science 10: 1617. Accessed October 20, 2023. https://www.frontiersin.org/articles/10.3389/fpls.2019.01617. 31921260 10.3389/fpls.2019.01617 PMC6920215

[ele70213-bib-0012] Caye, K. , B. Jumentier , J. Lepeule , and O. François . 2019. “LFMM 2: Fast and Accurate Inference of Gene‐Environment Associations in Genome‐Wide Studies.” Molecular Biology and Evolution 36, no. 4: 852–860. 10.1093/molbev/msz008. 30657943 PMC6659841

[ele70213-bib-0013] Chamberlain, S. A. , J. L. Bronstein , and J. A. Rudgers . 2014. “How Context Dependent Are Species Interactions?” Ecology Letters 17, no. 7: 881–890. 10.1111/ele.12279. 24735225

[ele70213-bib-0014] Chaudhary, V. B. , M. A. Rúa , A. Antoninka , et al. 2016. “MycoDB, a Global Database of Plant Response to Mycorrhizal Fungi.” Scientific Data 3, no. 1: 160028. 10.1038/sdata.2016.28. 27163938 PMC4862322

[ele70213-bib-0015] Cheng, S. , M. Melkonian , S. A. Smith , et al. 2018. “10KP: A Phylodiverse Genome Sequencing Plan.” GigaScience 7, no. 3: giy013. 10.1093/gigascience/giy013. 29618049 PMC5869286

[ele70213-bib-0017] Dobin, A. , C. A. Davis , F. Schlesinger , et al. 2013. “STAR: Ultrafast Universal RNA‐Seq Aligner.” Bioinformatics 29, no. 1: 15–21. 10.1093/bioinformatics/bts635. 23104886 PMC3530905

[ele70213-bib-0018] Donoghue, P. C. J. , and M. A. Purnell . 2005. “Genome Duplication, Extinction and Vertebrate Evolution.” Trends in Ecology & Evolution 20, no. 6: 312–319. 10.1016/j.tree.2005.04.008. 16701387

[ele70213-bib-0019] Emms, D. M. , and S. Kelly . 2015. “OrthoFinder: Solving Fundamental Biases in Whole Genome Comparisons Dramatically Improves Orthogroup Inference Accuracy.” Genome Biology 16, no. 1: 157. 10.1186/s13059-015-0721-2. 26243257 PMC4531804

[ele70213-bib-0020] Emms, D. M. , and S. Kelly . 2019. “OrthoFinder: Phylogenetic Orthology Inference for Comparative Genomics.” Genome Biology 20, no. 1: 238. 10.1186/s13059-019-1832-y. 31727128 PMC6857279

[ele70213-bib-0021] Fan, C. , Y. Chen , and M. Long . 2008. “Recurrent Tandem Gene Duplication Gave Rise to Functionally Divergent Genes in Drosophila.” Molecular Biology and Evolution 25, no. 7: 1451–1458. 10.1093/molbev/msn089. 18408233 PMC2878002

[ele70213-bib-0022] Fisher, K. J. , S. W. Buskirk , R. C. Vignogna , D. A. Marad , and G. I. Lang . 2018. “Adaptive Genome Duplication Affects Patterns of Molecular Evolution in *Saccharomyces cerevisiae* .” PLoS Genetics 14, no. 5: e1007396. 10.1371/journal.pgen.1007396. 29799840 PMC5991770

[ele70213-bib-0023] Freeling, M. 2009. “Bias in Plant Gene Content Following Different Sorts of Duplication: Tandem, Whole‐Genome, Segmental, or by Transposition.” Annual Review of Plant Biology 60, no. 1: 433–453. 10.1146/annurev.arplant.043008.092122. 19575588

[ele70213-bib-0024] Furumizu, C. , J. P. Alvarez , K. Sakakibara , and J. L. Bowman . 2015. “Antagonistic Roles for KNOX1 and KNOX2 Genes in Patterning the Land Plant Body Plan Following an Ancient Gene Duplication.” PLoS Genetics 11, no. 2: e1004980. 10.1371/journal.pgen.1004980. 25671434 PMC4335488

[ele70213-bib-0025] Garcia, K. , D. Chasman , S. Roy , and J. M. Ané . 2017. “Physiological Responses and Gene Co‐Expression Network of Mycorrhizal Roots Under K^+^ Deprivation.” Plant Physiology 173, no. 3: 1811–1823. 10.1104/pp.16.01959. 28159827 PMC5338680

[ele70213-bib-0027] Gralka, M. , R. Szabo , R. Stocker , and O. X. Cordero . 2020. “Trophic Interactions and the Drivers of Microbial Community Assembly.” Current Biology 30, no. 19: R1176–R1188. 10.1016/j.cub.2020.08.007. 33022263

[ele70213-bib-0028] Grigoriev, I. V. , R. Nikitin , S. Haridas , et al. 2014. “MycoCosm Portal: Gearing Up for 1000 Fungal Genomes.” Nucleic Acids Research 42, no. D1: D699–D704. 10.1093/nar/gkt1183. 24297253 PMC3965089

[ele70213-bib-0029] Hagen, M. , W. D. Kissling , C. Rasmussen , et al. 2012. “Biodiversity, Species Interactions and Ecological Networks in a Fragmented World.” In Advances in Ecological Research, 89–210. Elsevier. 10.1016/B978-0-12-396992-7.00002-2.

[ele70213-bib-0030] Hanada, K. , C. Zou , M. D. Lehti‐Shiu , K. Shinozaki , and S. H. Shiu . 2008. “Importance of Lineage‐Specific Expansion of Plant Tandem Duplicates in the Adaptive Response to Environmental Stimuli.” Plant Physiology 148, no. 2: 993–1003. 10.1104/pp.108.122457. 18715958 PMC2556807

[ele70213-bib-0031] Harrison, M. J. 2005. “Signaling in the Arbuscular Mycorrhizal Symbiosis.” Annual Review of Microbiology 59, no. 1: 19–42. 10.1146/annurev.micro.58.030603.123749. 16153162

[ele70213-bib-0032] Heckman, D. S. , D. M. Geiser , B. R. Eidell , R. L. Stauffer , N. L. Kardos , and S. B. Hedges . 2001. “Molecular Evidence for the Early Colonization of Land by Fungi and Plants.” Science 293, no. 5532: 1129–1133. 10.1126/science.1061457. 11498589

[ele70213-bib-0034] Hughes, G. M. , E. S. M. Boston , J. A. Finarelli , W. J. Murphy , D. G. Higgins , and E. C. Teeling . 2018. “The Birth and Death of Olfactory Receptor Gene Families in Mammalian Niche Adaptation.” Molecular Biology and Evolution 35, no. 6: 1390–1406. 10.1093/molbev/msy028. 29562344 PMC5967467

[ele70213-bib-0035] Jiang, H. , L. Xu , and Z. Gu . 2011. “Growth of Novel Epistatic Interactions by Gene Duplication.” Genome Biology and Evolution 3: 295–301. 10.1093/gbe/evr016. 21402864 PMC3274824

[ele70213-bib-0036] Levine, R. 2011. “i5k: The 5,000 Insect Genome Project.” American Entomologist 57, no. 2: 110–113. 10.1093/ae/57.2.110.

[ele70213-bib-0037] Li, H. 2011. “A Statistical Framework for SNP Calling, Mutation Discovery, Association Mapping and Population Genetical Parameter Estimation From Sequencing Data.” Bioinformatics 27, no. 21: 2987–2993. 10.1093/bioinformatics/btr509. 21903627 PMC3198575

[ele70213-bib-0039] Li, H.‐T. , T. S. Yi , L. M. Gao , et al. 2019. “Origin of Angiosperms and the Puzzle of the Jurassic Gap.” Nature Plants 5, no. 5: 461–470. 10.1038/s41477-019-0421-0. 31061536

[ele70213-bib-0041] Liu, O. R. , and S. D. Gaines . 2022. “Environmental Context Dependency in Species Interactions.” Proceedings of the National Academy of Sciences 119, no. 36: e2118539119. 10.1073/pnas.2118539119. PMC9457591 36037344

[ele70213-bib-0042] Love, M. I. , W. Huber , and S. Anders . 2014. “Moderated Estimation of Fold Change and Dispersion for RNA‐Seq Data With DESeq2.” Genome Biology 15, no. 12: 550. 10.1186/s13059-014-0550-8. 25516281 PMC4302049

[ele70213-bib-0043] Maherali, H. , B. Oberle , P. F. Stevens , W. K. Cornwell , and D. J. McGlinn . 2016. “Mutualism Persistence and Abandonment During the Evolution of the Mycorrhizal Symbiosis.” American Naturalist 188, no. 5: E113–E125. 10.1086/688675. 27788343

[ele70213-bib-0045] Mattenberger, F. , B. Sabater‐Muñoz , C. Toft , and M. A. Fares . 2017. “The Phenotypic Plasticity of Duplicated Genes in *Saccharomyces cerevisiae* and the Origin of Adaptations.” G3: Genes, Genomes, Genetics 7, no. 1: 63–75. 10.1534/g3.116.035329. 27799339 PMC5217124

[ele70213-bib-0046] Medicago HapMap Project . n.d. “Medicago HapMap Project.” https://medicago.legumeinfo.org/.

[ele70213-bib-0047] Monroe, J. G. , T. Powell , N. Price , et al. 2018. “Drought Adaptation in *Arabidopsis thaliana* by Extensive Genetic Loss‐of‐Function.” eLife 7: e41038. 10.7554/eLife.41038. 30520727 PMC6326724

[ele70213-bib-0048] Morris, J. L. , M. N. Puttick , J. W. Clark , et al. 2018. “The Timescale of Early Land Plant Evolution.” Proceedings of the National Academy of Sciences 115, no. 10: E2274–E2283. 10.1073/pnas.1719588115. PMC5877938 29463716

[ele70213-bib-0049] Müller, L. M. , K. Flokova , E. Schnabel , et al. 2019. “A CLE–SUNN Module Regulates Strigolactone Content and Fungal Colonization in Arbuscular Mycorrhiza.” Nature Plants 5, no. 9: 933–939. 10.1038/s41477-019-0501-1. 31477892

[ele70213-bib-0050] Noë, R. , and P. Hammerstein . 1995. “Biological Markets.” Trends in Ecology & Evolution 10, no. 8: 336–339. 10.1016/S0169-5347(00)89123-5. 21237061

[ele70213-bib-0051] Oldroyd, G. E. D. 2013. “Speak, Friend, and Enter: Signalling Systems That Promote Beneficial Symbiotic Associations in Plants.” Nature Reviews Microbiology 11, no. 4: 252–263. 10.1038/nrmicro2990. 23493145

[ele70213-bib-0053] Ponce, R. , and D. L. Hartl . 2006. “The Evolution of the Novel Sdic Gene Cluster in *Drosophila melanogaster* .” Gene 376, no. 2: 174–183. 10.1016/j.gene.2006.02.011. 16765537

[ele70213-bib-0054] Qiao, X. , Q. Li , H. Yin , et al. 2019. “Gene Duplication and Evolution in Recurring Polyploidization–Diploidization Cycles in Plants.” Genome Biology 20, no. 1: 38. 10.1186/s13059-019-1650-2. 30791939 PMC6383267

[ele70213-bib-0055] Qiao, X. , H. Yin , L. Li , R. Wang , J. Wu , and S. Zhang . 2018. “Different Modes of Gene Duplication Show Divergent Evolutionary Patterns and Contribute Differently to the Expansion of Gene Families Involved in Important Fruit Traits in Pear (*Pyrus bretschneideri*).” Frontiers in Plant Science 9: 161. 10.3389/fpls.2018.00161. 29487610 PMC5816897

[ele70213-bib-0056] R Core Team . 2025. R: A Language and Environment for Statistical Computing_. R Foundation for Statistical Computing. https://www.R‐project.org.

[ele70213-bib-0057] Ratzke, C. , J. Barrere , and J. Gore . 2020. “Strength of Species Interactions Determines Biodiversity and Stability in Microbial Communities.” Nature Ecology & Evolution 4, no. 3: 376–383. 10.1038/s41559-020-1099-4. 32042124

[ele70213-bib-0058] Recchia, G. H. , E. R. Konzen , F. Cassieri , D. G. G. Caldas , and S. M. Tsai . 2018. “Arbuscular Mycorrhizal Symbiosis Leads to Differential Regulation of Drought‐Responsive Genes in Tissue‐Specific Root Cells of Common Bean.” Frontiers in Microbiology 9: 1339. 10.3389/fmicb.2018.01339. 30013521 PMC6036286

[ele70213-bib-0059] Ren, C.‐G. , C. C. Kong , K. Yan , and Z. H. Xie . 2019. “Transcriptome Analysis Reveals the Impact of Arbuscular Mycorrhizal Symbiosis on *Sesbania cannabina* Expose to High Salinity.” Scientific Reports 9, no. 1: 2780. 10.1038/s41598-019-39463-0. 30808908 PMC6391373

[ele70213-bib-0060] Ren, R. , H. Wang , C. Guo , et al. 2018. “Widespread Whole Genome Duplications Contribute to Genome Complexity and Species Diversity in Angiosperms.” Molecular Plant 11, no. 3: 414–428. 10.1016/j.molp.2018.01.002. 29317285

[ele70213-bib-0061] Rogers, R. L. , J. M. Cridland , L. Shao , T. T. Hu , P. Andolfatto , and K. R. Thornton . 2015. “Tandem Duplications and the Limits of Natural Selection in *Drosophila yakuba* and *Drosophila simulans* .” PLoS One 10, no. 7: e0132184. 10.1371/journal.pone.0132184. 26176952 PMC4503668

[ele70213-bib-0062] Stanton‐Geddes, J. , T. Paape , B. Epstein , et al. 2013. “Candidate Genes and Genetic Architecture of Symbiotic and Agronomic Traits Revealed by Whole‐Genome, Sequence‐Based Association Genetics in *Medicago truncatula* .” PLoS One 8, no. 5: e65688. 10.1371/journal.pone.0065688. 23741505 PMC3669257

[ele70213-bib-0063] Strullu‐Derrien, C. , M. A. Selosse , P. Kenrick , and F. M. Martin . 2018. “The Origin and Evolution of Mycorrhizal Symbioses: From Palaeomycology to Phylogenomics.” New Phytologist 220, no. 4: 1012–1030. 10.1111/nph.15076. 29573278

[ele70213-bib-0066] Tank, D. C. , J. M. Eastman , M. W. Pennell , et al. 2015. “Nested Radiations and the Pulse of Angiosperm Diversification: Increased Diversification Rates Often Follow Whole Genome Duplications.” New Phytologist 207, no. 2: 454–467. 10.1111/nph.13491. 26053261

[ele70213-bib-0067] The Arabidopsis Genome Initiative . 2000. “Analysis of the Genome Sequence of the Flowering Plant *Arabidopsis thaliana* .” Nature 408: 796–815. 10.1038/35048692. 11130711

[ele70213-bib-0068] Venkatraman, M. , R. C. Fleischer , and M. T. N. Tsuchiya . 2021. “Comparative Analysis of Annotation Pipelines Using the First Japanese White‐Eye (*Zosterops japonicus*) Genome.” Genome Biology and Evolution 13: evab063. 10.1093/gbe/evab063. 33760049 PMC8120012

[ele70213-bib-0070] Voolstra, C. , D. J. Miller , M. A. Ragan , et al. 2015. “The ReFuGe 2020 Consortium—Using “Omics” Approaches to Explore the Adaptability and Resilience of Coral Holobionts to Environmental Change.” Frontiers in Marine Science 2: 68. Accessed November 10, 2022. https://www.frontiersin.org/articles/10.3389/fmars.2015.00068.

[ele70213-bib-0071] Walden, N. , D. A. German , E. M. Wolf , et al. 2020. “Nested Whole‐Genome Duplications Coincide With Diversification and High Morphological Disparity in Brassicaceae.” Nature Communications 11, no. 1: 3795. 10.1038/s41467-020-17605-7. PMC7393125 32732942

[ele70213-bib-0073] Wang, Y. , H. Tang , J. D. DeBarry , et al. 2012. “MCScanX: A Toolkit for Detection and Evolutionary Analysis of Gene Synteny and Collinearity.” Nucleic Acids Research 40, no. 7: e49. 10.1093/nar/gkr1293. 22217600 PMC3326336

[ele70213-bib-0074] Weisman, C. M. , A. W. Murray , and S. R. Eddy . 2022. “Mixing Genome Annotation Methods in a Comparative Analysis Inflates the Apparent Number of Lineage‐Specific Genes.” Current Biology 32, no. 12: 2632–2639.e2. 10.1016/j.cub.2022.04.085. 35588743 PMC9346927

[ele70213-bib-0075] Xie, T. , C. Chen , C. Li , J. Liu , C. Liu , and Y. He . 2018. “Genome‐Wide Investigation of WRKY Gene Family in Pineapple: Evolution and Expression Profiles During Development and Stress.” BMC Genomics 19, no. 1: 490. 10.1186/s12864-018-4880-x. 29940851 PMC6019807

[ele70213-bib-0076] Young, N. D. , and A. K. Bharti . 2012. “Genome‐Enabled Insights Into Legume Biology.” Annual Review of Plant Biology 63, no. 1: 283–305. 10.1146/annurev-arplant-042110-103754. 22404476

[ele70213-bib-0077] Young, N. D. , F. Debellé , G. E. D. Oldroyd , et al. 2011. “The Medicago Genome Provides Insight Into the Evolution of Rhizobial Symbioses.” Nature 480, no. 7378: 520–524. 10.1038/nature10625. 22089132 PMC3272368

[ele70213-bib-0078] Zhai, X. , D. Wu , C. Chen , et al. 2024. “A Chromosome Level Genome Assembly of Pseudoroegneria Libanotica Reveals a Key Kcs Gene Involves in the Cuticular Wax Elongation for Drought Resistance.” BMC Genomics 25, no. 1: 253. 10.1186/s12864-024-10140-5. 38448864 PMC10916072

